# Reference Gene Stability in *Agrostemma githago* Using Quantitative Real-Time PCR

**DOI:** 10.3390/ijms27020889

**Published:** 2026-01-15

**Authors:** Monika Bielecka, Bartosz Pencakowski, Marta Stafiniak, Weronika Kozłowska, Michał Dziwak, Katarzyna Nowis, Łukasz Łaczmański, Adam Matkowski

**Affiliations:** 1 Department of Pharmaceutical Biology and Biotechnology, Faculty of Pharmacy, Wroclaw Medical University, Borowska 211A, 50-556 Wroclaw, Polandweronika.kozlowska@umw.edu.pl (W.K.);; 2 Laboratory of Genomics & Bioinformatics, Hirszfeld Institute of Immunology and Experimental Therapy PAS, 53-114 Wroclaw, Poland; katarzyna.nowis@hirszfeld.pl (K.N.); lukasz.laczmanski@hirszfeld.pl (Ł.Ł.)

**Keywords:** housekeeping genes, RefFinder, qRT-PCR normalization, reference-gene validation, *Agrostemma githago*

## Abstract

Quantitative real-time PCR (qPCR) remains a cornerstone method for analyzing gene expression due to its high sensitivity, specificity, and reproducibility. However, for reliable results in relative quantification studies, the choice of an appropriate reference gene is critical to ensure accurate normalization. The expression of commonly used reference genes can vary depending on developmental stage and experimental conditions, making their validation essential. To date, no validated reference genes have been reported for *Agrostemma githago* L. (corn cockle, Caryophyllaceae). To facilitate research on genes involved in natural product biosynthesis and specialized metabolism regulation, we aimed to identify the most stable reference genes across various plant organs and cultivation conditions of this species. Drawing on previous literature, we have selected seven housekeeping genes widely used for evaluation: *actin*, *β-tubulin*, *elongation factor 1α*, *glyceraldehyde-3-phosphate dehydrogenase*, *histone H3*, *translation elongation factor 1*, and *eukaryotic translation initiation factor 5A1* (for which two primer sets were tested). The nucleotide sequences of these potential reference genes were identified from the *A. githago* transcriptome. Using qRT-PCR, transcript levels of seven potential reference genes were estimated in 40 different *A. githago* samples, including 25 in vitro samples under various treatment conditions and 15 soil-grown samples representing *A. githago* organs in different developmental stages. Expression stability of candidate reference genes was assessed using the RefFinder platform, which combines four commonly applied statistical algorithms: geNorm, NormFinder, BestKeeper, and the comparative Δ-Ct method. The results revealed that the selection of optimal reference genes varied based on the particular organ, developmental stage and condition being examined. *TIF5A1-2* (one of the two primer pairs tested) and *GAPHD* consistently exhibited the most stable expression under various conditions in vitro. *EF1α* and *H3* exhibited superior performance across different organs of soil-grown plants. Moreover, our integrated analysis enabled the identification of the two most stable, universal reference genes suitable for normalization in *A. githago* under all tested conditions—*H3* and *TIF5A1-2*. Our work provides a robust foundation for future transcriptomic and functional studies of the specialized metabolism of *A. githago* and other related species.

## 1. Introduction

Quantitative real-time PCR (qRT-PCR) remains one of the most widely used and reliable methods for quantifying gene expression, owing to its high sensitivity, specificity, reproducibility, and overall analytical efficiency [1,2]. However, the accuracy of qRT-PCR can be affected by factors such as sample source, RNA integrity, cDNA quality, primer specificity, amplification efficiency, and other experimental variables [3,4]. To minimize these sources of variation and ensure reliable measurements, it is essential to normalize raw expression data according to the Minimum Information for Publication of Quantitative Real-Time PCR Experiments (MIQE) guidelines [5,6] which emphasizes the selection of appropriate reference genes.

Reliable normalization requires reference genes with stable expression across tissues, developmental stages, and experimental conditions [7,8]. Housekeeping genes which maintain basic cellular functions and encode essential proteins are frequently used as reference genes, as their products are necessary for cellular activity and are typically expressed under a wide range of conditions. Traditionally, well-known housekeeping genes, such as *elongation factor 1 alpha* (*EF1α*), *glyceraldehyde-3-phosphate dehydrogenase* (*GAPDH*), *actin* (*ACT*) and *ubiquitin* (*UBQ*), were commonly accepted for normalization without prior validation of their stability. However, numerous studies have demonstrated that the transcript levels of many traditional housekeeping genes can vary substantially under different biological or environmental contexts as their expression stability is not universal and often species-, tissue-, or treatment-dependent [9,10]. Consequently, unstable reference genes can introduce substantial bias into qRT-PCR datasets [11,12].

Moreover, insights from RNA-seq data further revealed that individual members of housekeeping gene families often exhibit distinct tissue-specific expression profiles and differential responses to environmental stimuli [13,14,15,16,17]. Since qRT-PCR assays typically target single gene models, it is no longer acceptable to select conventional housekeeping genes arbitrarily, without prior confirming their expression stability across the specific samples and conditions under investigation [18]. Instead, rigorous, experiment-specific validation has become a necessary prerequisite for any reliable qRT-PCR-based gene expression study. Progress in high-throughput transcriptomics has also facilitated the identification of novel candidate reference genes with improved stability, identified through large-scale genome and transcriptome mining [7,14,15,16,19,20,21,22,23,24,25,26]. These studies consistently show that reference gene suitability is highly species- and condition-specific. Consequently, the systematic evaluation of known and novel candidate reference genes prior to experimentation is essential to ensure data accuracy and to avoid biased conclusions. The use of multiple reference genes, providing more robust normalization than any single gene alone, is nowadays recommended to reduce systematic error and enhance data reliability [27,28]. The diverse physiological and ecological adaptations of non-model species, such as many medicinal and poisonous plants, also mean that genes involved in basic cellular functions might be regulated differently under various environmental stresses or developmental stages, further complicating the search for true expression stability [29,30]. Hence, the identification of stable reference genes presents unique challenges compared to well-characterized model organisms. This underscores the need for systematic, empirical validation of reference genes tailored to the specific experimental context of each non-model plant.

The plant used in this study, *Agrostemma githago* L. (corn cockle), Caryophyllaceae, is an annual herbaceous segetal weed that was once widespread in cereal fields, where its seeds often contaminated harvested grain. However, it has become increasingly rare due to modern agricultural practices. Nowadays, it is considered endangered in many parts of Europe and has emerged as an ornamental plant for use in wildflower meadows, and a few new varieties have been bred. Despite its decline, *A. githago* remains ecologically and evolutionarily significant, representing a unique lineage adapted to variable environmental conditions under anthropogenic pressure, such as seed persistence. Moreover, the historical and recent reports on its medicinal and toxic properties of this understudied plant call for a comprehensive characterization of its functional genomics.

Previous studies have revealed the presence of a number of triterpene saponins [31], and type I ribosome-inactivating proteins (RIPs) in *A. githago* [32]. Some of the saponins from this species, such as agrostemmoside E, have the capacity to facilitate the endosomal escape of type I RIPs within the cell, a critical step for RIP-mediated toxicity [33,34]. A comprehensive review of phytochemical and bioactive properties of corncockle has been published recently [35]. Furthermore, while the genome sequence of *A. githago* has recently become available [36], the molecular regulation of saponin biosynthesis and the expression of type I RIPs have not yet been reported.

Despite its biochemical, pharmacological, ecological, and evolutionary interest, a robust framework for gene expression analysis in *A. githago*, particularly regarding the identification of reliable reference genes for qRT-PCR, remains lacking, as no validated reference genes have been reported for this species to date. However, within the Caryophyllaceae family, a few studies have investigated the stability of potential reference genes in species such as *Silene vulgaris* [37], *Dianthus caryophyllus* [38], and *Dianthus broteri* [39]. To support investigations of genes involved in specialized metabolism underlying natural product biosynthesis and the regulation of RIP production in *A. githago*, we sought to identify reference genes exhibiting the highest expression stability across diverse organs and cultivation conditions of this species. Based on previous studies in carnation species, we selected seven candidate reference genes for investigation: *actin*, *β-tubulin*, *elongation factor 1α*, *glyceraldehyde-3-phosphate dehydrogenase*, *histone H3*, *translation elongation factor 1*, and *eukaryotic translation initiation factor 5A1*. Orthologous nucleotide sequences of these genes were retrieved from the transcriptomic data of *A. githago* to enable primer design [32]. Using qRT-PCR, transcript levels of these seven potential reference genes were estimated in 40 different *A. githago* samples, including 25 in vitro samples under various treatment conditions and 15 soil-grown samples representing different organs and developmental stages. Expression stability was evaluated from Ct values using the comparative ΔCt method [40], geNorm [41], NormFinder [42], BestKeeper [43], and the integrative RefFinder algorithm [44,45], and their accuracy was validated by analyzing the expression of the *Squalene synthase (SQS)* and *β-amyrin synthase (BAS)* genes.

This study is the first to screen and verify the internal reference genes used for qRT-PCR normalization in *A. githago* across various plant organs and cultivation conditions. To support downstream gene expression analyses in *A. githago*, we leveraged existing knowledge from Caryophyllaceae, selected previously validated candidate reference genes, and systematically assessed their stability under our experimental conditions. The main strength of this study lies in the rigorous evaluation of these candidates across 40 independent *A. githago* cDNA libraries spanning diverse developmental stages and cultivation regimes. This application-oriented work builds on prior research to provide a practical framework for selecting appropriate reference genes for *A. githago* grown either in soil or in vitro.

By identifying universally stable housekeeping genes, we also provide valuable resources for future quantification of gene expression in *A. githago* and related species, facilitating studies not only of specific biosynthetic pathways but also of broader physiological and ecological processes.

## 2. Results

### 2.1. Primer Specificity and Amplification Efficiency

**Figure 1:**
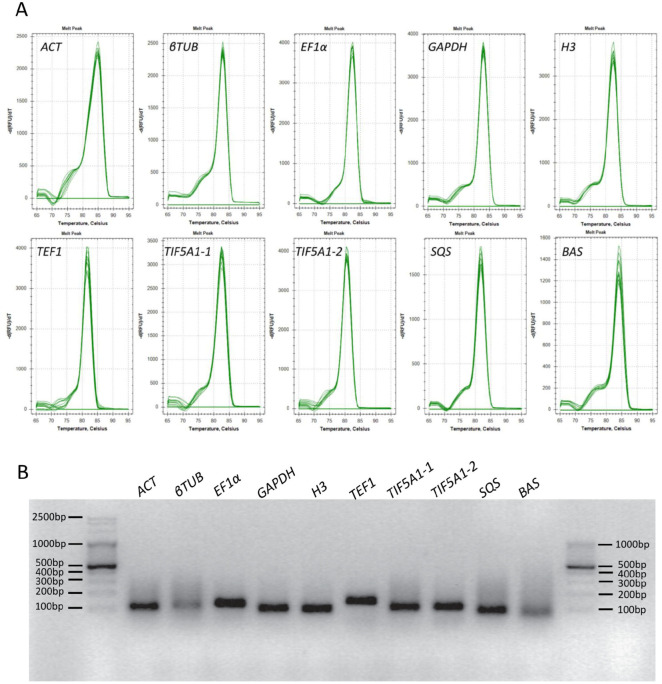
Primer specificity of candidate reference genes verified by melting curve analysis shown using Bio-Rad CFX Maestro 1.1 software (version 4.1.2433.1219) (**A**) and 2% agarose gel electrophoresis (**B**). Amplicon sizes were estimated against mass markers: Perfect^TM^ 100 bp DNA Ladder (EURx, Gdansk, Poland) (**left**) and Perfect^TM^ 100–1000 bp DNA Ladder (EURx, Gdansk, Poland) (**right**).

Primer specificity and amplification efficiency (E) were confirmed through melting and standard-curve analysis, respectively. As depicted in Figure 1A, melt-curve analysis revealed a single peak for each candidate reference gene, indicating highly specific amplification. Agarose gel electrophoresis confirmed that each primer pair produced a single amplicon within the expected size range (86–149 bp) (Figure 1B). No non-specific bands or primer dimers were detected.

Amplification efficiencies ranged from 96% (*H3*) to 117% (*TIF5A1-1*), while correlation coefficients (R^2^) values spanned 0.991–0.999. These results demonstrated that all primer sets have suitable specificity and efficiency for subsequent qRT-PCR analyses. Primer sequences, amplicon lengths, empirically established efficiencies, and R^2^ values are provided in Table 1.

All candidate reference gene primers exhibited high specificity and acceptable amplification performance, confirming their suitability for qRT-PCR analyses.

### 2.2. Expression Profiles of Candidate Reference Genes

The expression consistency of the seven candidate reference genes across *A. githago* organs and under diverse growth and stress conditions was evaluated based on cycle threshold (Ct) values obtained via qRT-PCR. As Ct values are inversely related to transcript abundance, lower Ct values correspond to higher gene expression.

Across all samples, Ct values ranged from 17.61 to 34.48. Mean Ct values spanned from 19.43 for *EF1α*, indicating its highest transcript abundance, to 30.39 for *TEF1*, reflecting comparatively low expression. This overall pattern was consistent when in vitro and soil-grown samples were analyzed independently (Supplementary Table S1).

**Figure 2:**
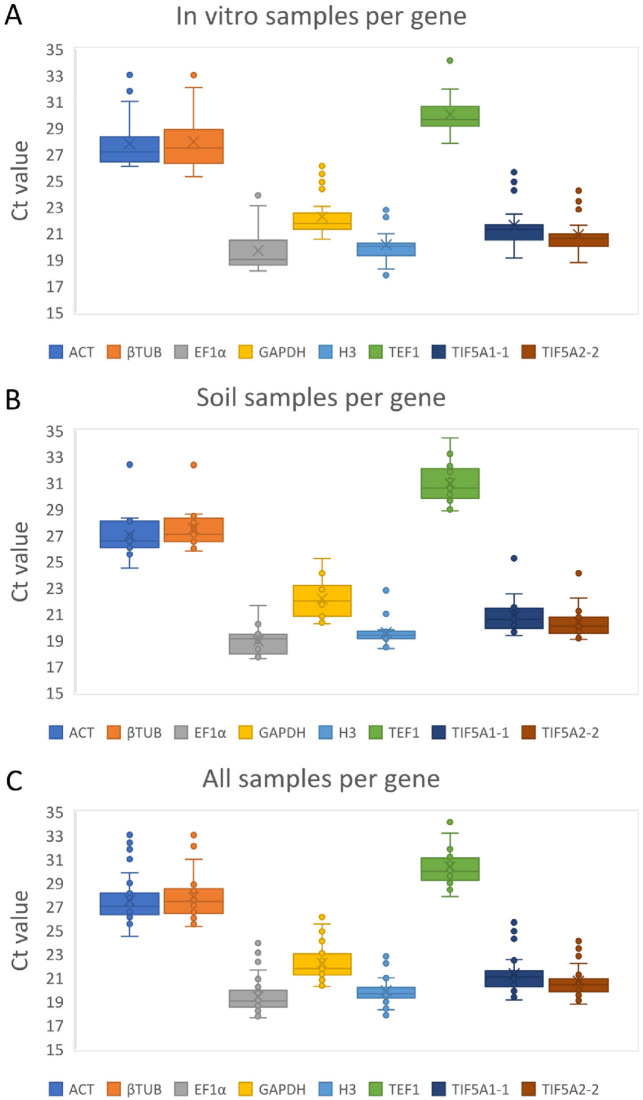
Expression levels of seven candidate reference genes across in vitro and soil cultivated *A. githago*, presented as cycle threshold (Ct) values. Distribution of mean Ct values of seven genes (represented by eight primer pairs) per in vitro cultured experimental samples (**A**), in soil-grown samples (**B**) and in all *A. githago* samples (**C**). For each boxplot, the average (diagonal cross), median (horizontal line), interquartile range (boxes: 25th to 75th percentile), and range (whiskers: minimum to maximum) are displayed, illustrating variation in transcript abundance across samples.

Box-plot visualizations of Ct distributions (Figure 2) illustrate expression variation among samples. Genes exhibiting narrow Ct dispersion were considered more stable. *H3* and *TIF5A1-2* displayed the lowest variation, indicating highly consistent expression across all tested conditions, whereas *βTUB*, *GAPDH*, and *TEF1* showed the greatest variability.

Within in vitro cultures, *H3*, followed by *TIF5A1-2* and *TIF5A1-1*, demonstrated the highest expression stability. In contrast, *βTUB* and *ACT* were the least stable and, together with *GAPDH*, exhibited the highest Ct values. In soil-grown plants, *H3* again showed the greatest expression stability, while *TEF1* and *GAPDH* displayed broader Ct dispersion, indicating reduced stability. Notably, *TEF1* consistently produced the highest Ct values among all candidate genes, clearly distinguishing it as the least abundantly expressed.

Our results show that genes traditionally regarded as stably expressed exhibit substantial differences in transcript abundance under the conditions of our experiment. Across all organs and experimental conditions, *H3* and *TIF5A1-2* exhibited the most consistent Ct values and highest expression stability, whereas *βTUB*, *GAPDH*, and *TEF1* showed substantial variability, with *TEF1* consistently displaying the lowest transcript abundance and poorest stability.

### 2.3. Expression Stability Assessment

Given substantial variation in expression levels among candidate reference genes across samples, statistical analyses were used to assess and rank their expression stability and determine the optimal number of reference genes required for accurate normalization in gene expression studies.

#### 2.3.1. ΔCt Method

**Figure 3:**
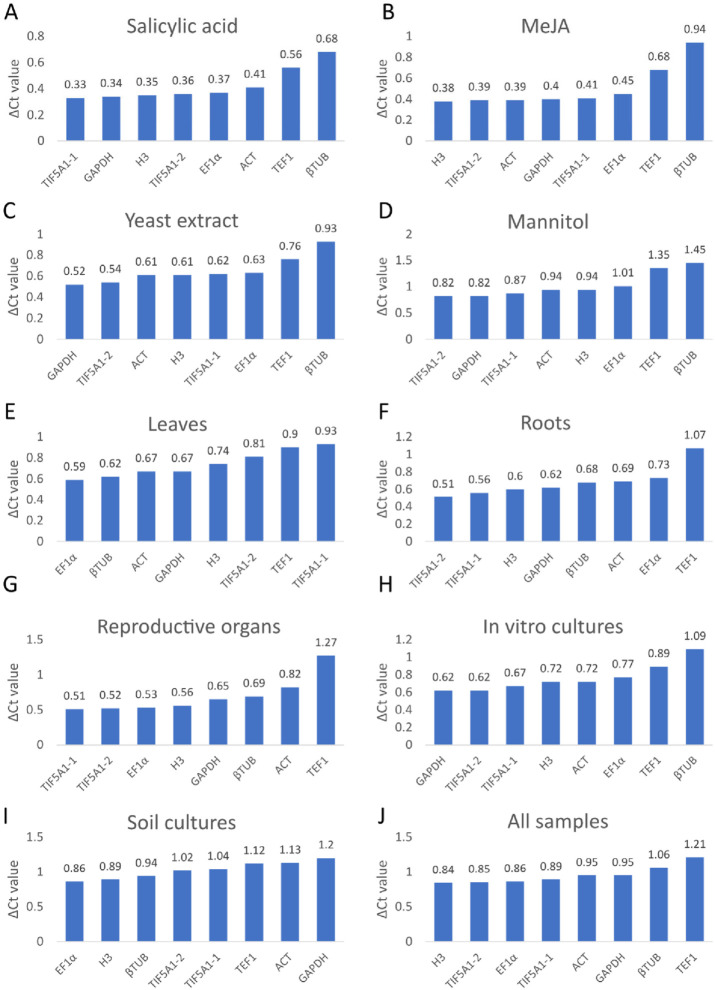
Stability evaluation of seven reference genes analyzed using ∆Ct. In vitro cultured *A. githago* treated with phytohormones: salicylic acid (**A**) and methyl jasmonate (**B**), under a biotic stress with yeast extract (**C**), under the osmotic stress with mannitol (**D**), in leaves (**E**), roots (**F**) and reproductive organs (**G**) of soil-grown plants, in all in vitro cultivated samples (**H**), in all soil-grown samples (**I**), and in all samples (**J**). Genes with the lowest average standard deviation across samples are considered the most stable.

The stability of each candidate reference gene was evaluated using the ΔCt method, in which genes with the lowest average standard deviation across samples are considered the most stable [37]. As shown in Figure 3, differences in stability rankings among successive genes were relatively small, indicating generally high stability across the dataset, although certain genes displayed notably lower variability than others.

Across all *A. githago* samples, *H3* showed the least expression variation and was therefore identified as the most stable reference gene, followed by *TIF5A1-2* and *EF1α*. In soil-grown plants, *TIF5A1-1* and *TIF5A1-2* were the most stable in roots and reproductive organs, whereas in leaves *EF1α* and *βTUB* exhibited the lowest variability. In contrast, within in vitro cultures, *GAPDH* and *TIF5A1-1*, followed closely by *TIF5A1-2*, produced the lowest ΔCt values, indicating the highest stability under these conditions.

Stability patterns also varied across treatment regimes. *TIF5A1-1* was the most stable under salicylic acid (SA) treatment, *H3* under methyl jasmonate (MeJA) treatment, *GAPDH* under yeast extract (YE) treatment, and *TIF5A1-2* under mannitol (MN) treatment. Notably, mannitol-treated samples, as well as soil-grown samples overall, exhibited greater variability in ΔCt values than other groups.

Using the ΔCt method, *H3* emerged as the most stable reference gene across all *A. githago* samples, with stability rankings varying by organ, growth condition, and treatment—highlighting context-dependent performance of candidate genes despite generally high overall expression stability.

#### 2.3.2. GeNorm Analysis

**Figure 4:**
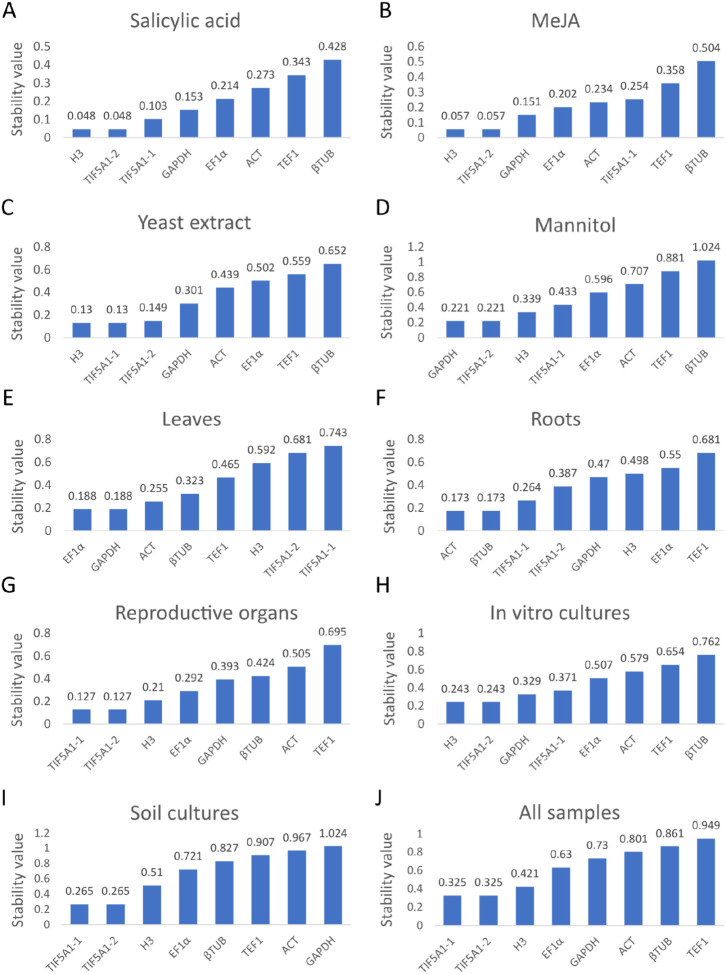
The ranking order of reference genes as expression stability values (M) of candidate reference genes calculated by geNorm. In vitro cultured *A. githago* treated with phytohormones: salicylic acid (**A**) and methyl jasmonate (**B**), under a biotic stress with yeast extract (**C**), under the osmotic stress with mannitol (**D**), in leaves (**E**), roots (**F**) and reproductive organs (**G**) of soil-grown plants, in all in vitro cultivated samples (**H**), in all soil-grown samples (**I**), and in all samples (**J**). Lower stability values indicate higher expression stability; values below 1.5 are considered acceptable for reference gene selection.

The stability of the seven candidate reference genes was evaluated using the geNorm algorithm, which calculates an average expression stability value (M) based on pairwise comparisons among all genes. Lower M values correspond to greater expression stability, and values below 1.5 are generally considered acceptable for reference gene selection [38]. In this investigation, all genes demonstrated M values below this threshold across all tested conditions, confirming their suitability as reference genes in *A. githago* (Figure 4). M values were lowest in in vitro samples; however, even in soil-grown plants, all values remained below 1.5, further supporting the appropriateness of the selected candidates and validating their stability as demonstrated by the geNorm analysis.

geNorm analysis showed that all seven candidate reference genes exhibited acceptable expression stability (M < 1.5) across all tested conditions, confirming their suitability for qRT-PCR normalization in *A. githago*.

In addition to ranking gene stability, geNorm was also used to determine the optimal number of reference genes required for accurate normalization. This analysis relies on calculating pairwise variation (Vn/Vn+1) between consecutive normalization factors (NF). A commonly accepted threshold of V < 0.15 is used to determine whether including an additional reference gene provides a meaningful improvement in normalization accuracy.

**Figure 5:**
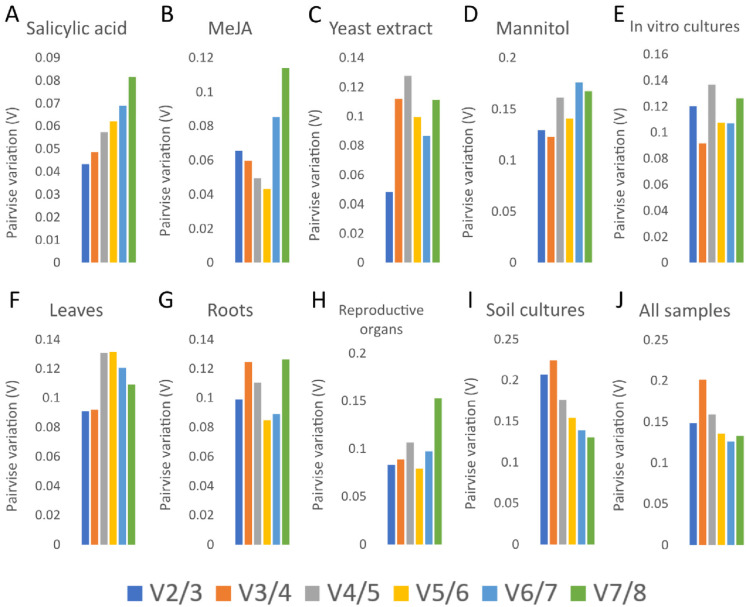
Pairwise variation (V) of seven candidate reference genes calculated by geNorm. Vn/Vn+1 values were used to determine the optimal number of reference genes; a threshold of V < 0.15 is used to assess whether inclusion of an additional reference gene results in a meaningful improvement in normalization accuracy. In vitro cultured *A. githago* treated with phytohormones: salicylic acid (**A**) and methyl jasmonate (**B**), under a biotic stress with yeast extract (**C**), under the osmotic stress with mannitol (**D**), in all in vitro cultivated samples (**E**), in leaves (**F**), roots (**G**) and reproductive organs (**H**) of soil-grown plants, in all soil-grown samples (**I**), and in all samples (**J**).

The analysis of all 40 cDNA samples indicated that including a third reference gene would not substantially enhance normalization accuracy, as the pairwise variation value V2/3 was 0.149, which meets the cut-off (<0.15) (Figure 5) (Supplementary Table S2). For the remaining analyses—across individual organs and under various growth and stress conditions—a similar trend was observed, indicating that adding a third reference gene would not meaningfully improve normalization accuracy. An exception occurred in the analysis of all soil-grown plants, where V5/6 = 0.154 and V6/7 = 0.139. These values suggest that six reference genes are adequate, as the inclusion of the seventh gene provides only a marginal improvement in normalization.

#### 2.3.3. NormFinder Analysis

To validate the geNorm results, the stability of the candidate reference genes was re-assessed using NormFinder. This model-based algorithm calculates an expression stability value (S) for each gene by quantifying both intra- and inter-group variation [39]. Prior to analysis, Ct values are transformed into relative expression levels, as required by the method. Lower S values correspond to higher expression stability and thus greater suitability as reference genes. In contrast to geNorm, which is based on pairwise comparisons, NormFinder uses an analysis of variance (ANOVA) framework to estimate variability and directly identify the most stable genes across defined sample groups.

**Figure 6:**
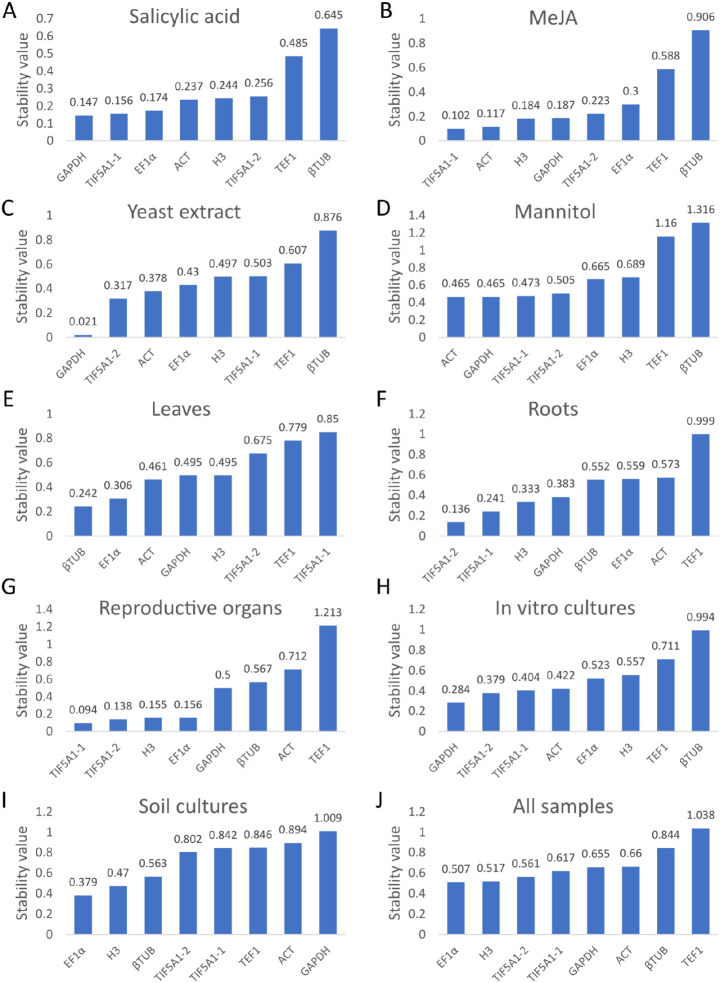
The ranking order of reference genes as expression stability values (M) of candidate reference genes calculated by NormFinder. In vitro cultured *A. githago* treated with phytohormones: salicylic acid (**A**) and methyl jasmonate (**B**), under a biotic stress with yeast extract (**C**), under the osmotic stress with mannitol (**D**), in leaves (**E**), roots (**F**) and reproductive organs (**G**) of soil-grown plants, in all in vitro cultivated samples (**H**), in all soil-grown samples (**I**), and in all samples (**J**). Lower stability values indicate higher expression stability.

The NormFinder analysis revealed substantial variation in reference gene performance across tissues and experimental conditions. When all samples were evaluated collectively, *EF1α* (S = 0.507) and *H3* (S = 0.517) were identified as the most stable genes, indicating their suitability as broadly applicable reference genes (Figure 6). In leaf tissue, *βTUB* (S = 0.242) and *EF1α* (S = 0.306) exhibited the highest stability, whereas in roots, *TIF5A1-2* and *TIF5A1-1* (S = 0.136 and 0.241, respectively) were the most stable. A similar pattern was observed in reproductive organs, where *TIF5A1-2* and *TIF5A1-1* again ranked highest (S = 0.138 and 0.094), both showing exceptionally low S values.

Across all in vitro samples, *GAPDH* (S = 0.284) showed the greatest stability, followed closely by *TIF5A1-2* (S = 0.379) and *TIF5A1-1* (S = 0.404). Under SA treatment, *GAPDH* (S = 0.147) and *TIF5A1-1* (S = 0.156) were the most stable, while *TIF5A1-1* (S = 0.102) and *ACT* (S = 0.117) ranked highest under MeJA treatment. In yeast extract–treated samples, *GAPDH* (S = 0.021) and *TIF5A1-2* (S = 0.317) were the most stable. In contrast, under mannitol stress, both *ACT* and *GAPDH* exhibited relatively high S values (S = 0.465), suggesting reduced stability in this condition.

Notably, *βTUB* consistently demonstrated poor stability across in vitro samples, and *TEF1* across different organs in soil-grown plants, indicating that these genes are less suitable as reference genes for normalization in *A. githago* according to the NormFinder analysis.

NormFinder analysis revealed pronounced tissue- and condition-dependent differences in reference gene stability, identifying *EF1α* and *H3* as the most stable overall, while highlighting *TIF5A1-1*, *TIF5A1-2*, and *GAPDH* as condition-specific top performers and confirming *βTUB* and *TEF1* as the least suitable reference genes in *A. githago*.

#### 2.3.4. BestKeeper Analysis

BestKeeper evaluates reference gene stability through the calculation of standard deviation (SD) and coefficient of variation (CV) of Ct values, where lower values denote greater stability. In addition, it computes Pearson correlation coefficients (r) between each candidate gene and the BestKeeper index, defined as the geometric mean of all tested reference genes. Genes are ranked by increasing SD, with SD ≤ 1 indicating high expression stability [40]. This descriptive statistical approach provides a complementary perspective to geNorm and NormFinder, offering additional validation for the selection of stable reference genes.

**Figure 7:**
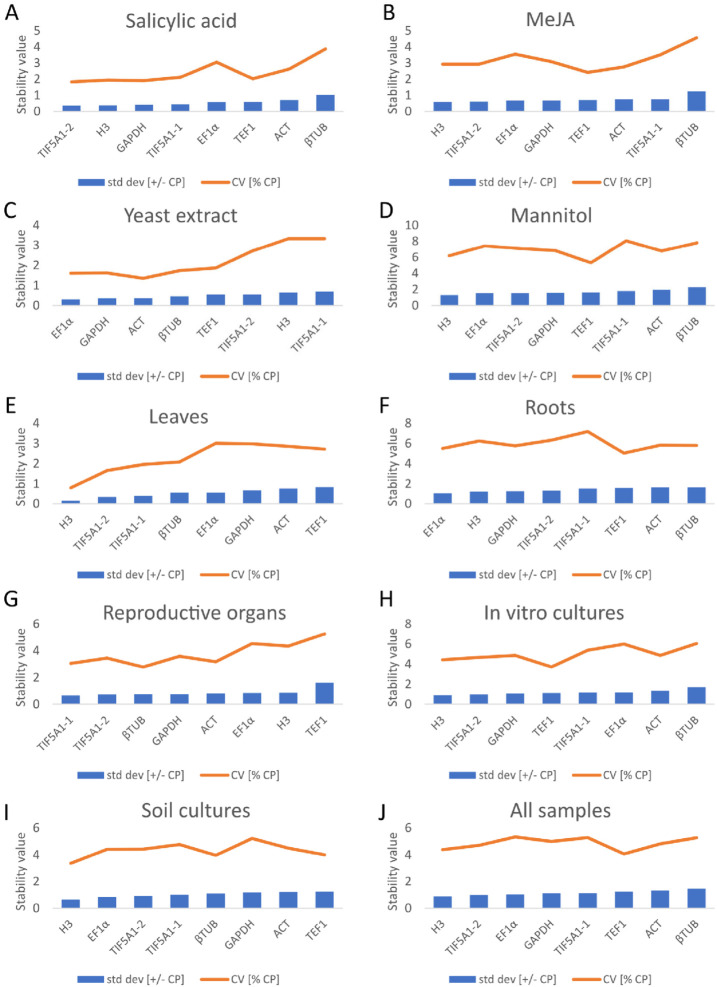
Stability evaluation of reference genes calculated by BestKeeper; with SD ≤ 1 indicating high expression stability. In vitro cultured *A. githago* treated with phytohormones: salicylic acid (**A**) and methyl jasmonate (**B**), under a biotic stress with yeast extract (**C**), under the osmotic stress with mannitol (**D**), in leaves (**E**), roots (**F**) and reproductive organs (**G**) of soil-grown plants, in all in vitro cultivated samples (**H**), in all soil-grown samples (**I**), and in all samples (**J**).

The BestKeeper analysis revealed that *H3* (SD = 0.88; CV = 4.39%) and *TIF5A1-2* (SD = 0.98; CV = 4.71%) were the most stable genes overall (Figure 7) (Supplementary Table S3). *EF1α* also showed low variability (SD = 1.03; CV = 5.33%), supporting its suitability for normalization. In contrast, across all samples, *βTUB* (SD = 1.46; CV = 5.27%) and *ACT* (SD = 1.33; CV = 4.82%) were identified as the least stable genes.

Gene stability varied considerably across soil-grown tissues. In leaf samples, *H3* (SD = 0.15; CV = 0.8%) and *TIF5A1-2* (SD = 0.33; CV = 1.66%) exhibited the highest stability, although all genes met the stability criterion (SD ≤ 1). In roots, *EF1α* (SD = 1.06; CV = 5.51%) was the most stable, yet still borderline, and the remaining genes showed higher variability and may be unsuitable for normalization. In reproductive organs, *TIF5A1-1* (SD = 0.66; CV = 3.05%) ranked as the most stable gene, whereas only *TEF1* exceeded the SD cut-off.

Stress-specific analyses further highlighted the importance of condition-dependent selection of reference genes. *H3* and *TIF5A1-2* were the most stable genes under salicylic acid and methyl jasmonate treatments, while *βTUB* was consistently the least stable (SD > 1). Under yeast extract treatment, all genes displayed strong stability, with the best performance from *EF1α* (SD = 0.3; CV = 1.6%) and *GAPDH* (SD = 0.35; CV = 1.62%). In contrast, mannitol-stressed samples showed the highest variability, with *H3* exhibiting the lowest stability values (SD = 1.29; CV = 6.19%).

Overall, the BestKeeper results demonstrate that reference gene stability is strongly influenced by tissue type, growth conditions, and stress treatment. *TIF5A1-2*, *GAPDH*, *H3*, and *EF1α* consistently ranked among the most stable genes across multiple conditions and are therefore recommended for normalization, whereas *βTUB* and *TEF1* showed high variability and should be avoided, according to BestKeeper analysis.

#### 2.3.5. Comprehensive Stability Ranking

Across all samples, *H3* was identified as the most stable reference gene (Geomean = 1.57), making it the strongest candidate for general normalization across different *A. githago* tissues and growth conditions (Table 2; Supplementary Table S4). It was closely followed by *TIF5A1-2* (1.86), which also serves as a reliable option for broad-scale expression studies. In contrast, *βTUB* (7.24) and *TEF1* (7.44) showed the lowest stability overall and should be avoided due to their inconsistent expression profiles.

In soil-grown plants, *EF1α* (1.68) and *H3* (1.86) were ranked as the most stable genes, while *GAPDH*, *ACT*, and *TEF1* (7.44, 7.24, and 6.45, respectively) showed poor stability under these conditions. Organ-specific analyses produced slightly different stability rankings: *EF1α* (1.78) demonstrated the highest stability in leaves, while *TIF5A1-1* (1.0) and *TIF5A1-2* (2.0) were the most stable in reproductive organs and roots. However, all tissue-specific analyses consistently identified *TEF1* as the least stable gene, confirming its unsuitability for normalization in soil-cultivated plants.

In in vitro cultures and under various stress treatments, *TIF5A1-1*, *TIF5A1-2*, *H3*, and *GAPDH* were identified as the most reliable reference genes across the analyses. Conversely, *βTUB* consistently exhibited the lowest stability, thereby reaffirming its exclusion from normalization under these conditions.

Overall stability analyses consistently identified *H3* and *TIF5A1-2* (together with *EF1α* in soil-grown plants and *TIF5A1-1*/*GAPDH* for in vitro and stress conditions) as the most reliable reference genes for qRT-PCR normalization in *A. githago*, whereas *βTUB* and *TEF1* showed poor stability across multiple datasets and therefore should be avoided as general normalizers.

### 2.4. Evaluation of Candidate Reference Genes for Expression Analysis in A. githago

To evaluate the reliability of the selected reference genes, we examined the expression patterns of *squalene synthase* (*SQS*) and *β-amyrin synthase* (*BAS*) across different experimental stress conditions and various organs of soil-grown plants.

These genes encode essential enzymes involved in triterpene and sterol biosynthesis, facilitating key steps in transforming farnesyl pyrophosphate into complex plant metabolites. *SQS* catalyzes the initial committed step, condensing two farnesyl pyrophosphate molecules to produce squalene A [41]. *β-amyrin synthase* then converts squalene-derived intermediates into β-amyrin, a precursor of triterpene saponins [42].

These enzymes can be induced by elicitors like methyl jasmonate, indicating they have adaptive functions in plant metabolism [43].

Based on RefFinder rankings, the two most stable and one most unstable reference genes were used to normalize *SQS* and *BAS* expression data. The performance of these reference genes was evaluated to determine their suitability for accurate gene expression analysis in different growth conditions.

**Figure 8:**
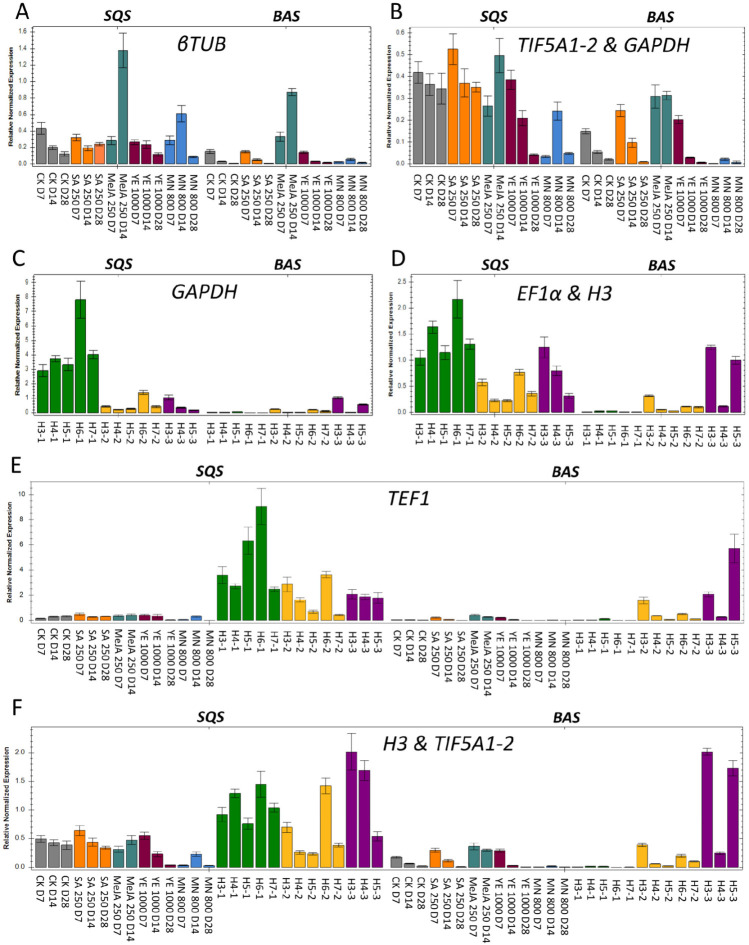
Relative expression levels of target genes *SQS* and *BAS* in *A. githago* cultivated in vitro (**A**,**B**), in soil-grown (**C**,**D**) and in all plant samples (**E**,**F**). A combination of the most stable reference genes (**B**,**D**,**F**) and the most unstable gene (**A**,**C**,**E**) were selected for normalization. Error bars show the standard error calculated from three biological replicates. CK, control; SA, salicylic acid; MeJA, methyl jasmonate; YE, yeast extract; MN, mannitol; D7, day 7; D14, day 14; D28, day 28; H, harvest. H3-1–H7-1—leaves; H3-2–H7-2—roots; H3-3–H5-3—reproductive organs.

As shown in Figure 8, the expression patterns of *SQS* and *BAS* were consistent and biologically reasonable when normalized using the stable reference genes. In contrast, normalization with the unstable reference genes, resulted in markedly altered expression profiles characterized by inflated variability between plant organs and various conditions and inconsistent trends. These discrepancies underscored the importance of reference gene stability in interpreting qPCR data. The results highlighted that the use of unreliable reference genes can distort target gene expression and compromise experimental accuracy, further validating the selection of *H3 and TIF5A1-2* as appropriate normalizers in *A. githago*.

## 3. Discussion

Housekeeping genes are traditionally defined by evolutionary conservation, stable expression, involvement in essential cellular functions and core maintenance processes, and thus were assumed to be expressed regardless of tissue, developmental stage, or environmental condition [44]. However, many studies have shown that the expression of housekeeping genes varies across tissues, developmental stages, treatments, environmental contexts, and species [45,46,47]. Consequently, rigorous evaluation of reference gene stability is crucial for reliable qPCR normalization [46,48] and the use of multiple validated reference genes is increasingly recommended to enhance the accuracy of gene expression analyses [28,49].

Relying on prior results published for Caryophyllaceae [50,51,52], we have selected seven housekeeping genes to study their expression stability in *A. githago*. We have omitted genes with insufficiently supported homology among the contig sequences of *A. githago* transcriptome [32]. We also excluded the gene encoding the 18S rRNA subunit from our analysis, as our goal was to identify universal corn cockle reference genes that are not influenced by variations in the rRNA to mRNA ratio across samples or experimental groups and that remain suitable for normalization regardless of the RNA purification method or cDNA synthesis protocol [10,52].

In this study, we assessed the expression stability of seven putative reference genes across multiple organs and cultivation conditions of *A. githago* using four widely employed algorithms: ΔCt, geNorm, NormFinder, and BestKeeper. To obtain an integrated ranking, the outputs of these individual methods were consolidated with the RefFinder tool, which computes a weighted geometric mean to generate a consensus assessment. By combining multiple statistical approaches, this strategy minimizes method-specific biases and yields a more reliable evaluation of reference gene performance.

Each algorithm offered distinct perspectives on reference gene performance. The ΔCt approach, which evaluates the uniformity of Ct differences between gene pairs across samples, identified *H3* as the most consistently expressed candidate in all datasets, followed closely by *TIF5A1-2* and *EF1α*. This result underscores its strong suitability as a broadly applicable reference gene for *A. githago*. The geNorm algorithm, which ranks candidates based on pairwise comparisons and calculates M-values, confirmed the suitability of all evaluated genes as appropriate normalizers in all tested organs and conditions. This general good performance of housekeeping genes tested in this work might result from earlier preselection or gene candidates, which has been performed based on published data in Caryophyllaceae [50,51,52]. However, when the geNorm was used to determine the optimal number of reference genes required for an accurate normalization, a minimal number of two genes was indicated, when analyzing all 40 cDNA samples. Except for soil-cultivated plants, it was proved that adding a third reference gene would not meaningfully improve normalization accuracy. Reassessment of the candidate genes stability performed by NormFinder revealed substantial variation in their performance across plant organs and cultivation conditions, however, collective evaluation of all 40 samples identified the *EF1α* and *H3* as the most stable. Complementary analysis done by BestKeeper revealed the highest expression stability of *H3* and *TIF5A1-2* followed by *EF1α*, indicating their suitability as broadly applicable reference genes.

A combined ranking from four algorithms presented by RefFinder identified *H3* as the most stable reference gene across all analysed *A. githago* samples. A similarly high level of stability was observed for *TIF5A1-2*, underscoring its potential as an additional dependable reference gene for comprehensive expression analyses in corn cockle. In contrast, *βTUB* and *TEF1* showed the lowest stability overall and are not recommended as suitable normalizers.

The ranking of genes, however, differed when performed for selected groups of samples. Across in vitro cultures and multiple stress treatments, *TIF5A1-1*, *TIF5A1-2*, *H3*, and *GAPDH* consistently emerged as the most stable reference genes. In contrast, *βTUB* showed the poorest stability in all in vitro analyses, confirming that it is unsuitable for normalization under these conditions. In soil-grown plants, *EF1α* and *H3* were ranked as the most stable genes, while *GAPDH*, *ACT*, and *TEF1* were the least stable and hence unsuitable for normalization in soil-grown plants. Notably, *GAPDH* expression variability changed drastically depending on the cultivation conditions, resulting in either high or poor suitability of this gene as a reference in in vitro or soil-based experiments, respectively.

Based on the comprehensive ranking, we propose to employ the combination of *TIF5A1-2* and *GAPDH* as reliable reference genes in in vitro experiments and the combination of the *EF1α* and *H3* in the applications were various organs and developmental stages are of interest in the soil-cultivated *A. githago*. As universal reference genes in corn cockle we propose the combination of *H3* and *TIF5A1-2* as these two genes presented superior stability in terms of expression level across all analysed *A. githago* samples, regardless of the plant organ, developmental stage and cultivation conditions. Our analysis confirmed and aligns with previous recommendations that distinct reference genes should be selected for different sample types and experimental conditions [27,28].

To validate the selected reference genes, we analyzed the expression profiles of SQS and BAS, two key enzymes in the triterpenoid saponin pathway. Normalization with the top-ranked reference genes, whether suitable for the specific cultivation conditions or universal, yielded consistent and biologically meaningful expression patterns across plant organs and experimental conditions. In contrast, using unstable reference genes generated irregular profiles and inflated variability. These results again confirm and are in line with previous findings that an inappropriate reference gene choice might lead to a biased understanding of qRT-PCR results [30] and mask the true nature of gene expression [53].

Additionally, the gene identity of the transcriptome-derived contigs [32] has been confirmed and linked with the recently annotated reference genome of *A. githago* (assembly dcAgrGith1.1) [36]. Several candidate reference genes mapped to multiple highly similar transcripts in the dcAgrGith1.1 genome assembly, frequently exhibiting near-complete sequence identity (Supplementary Table S7). Notably, many of these alignments correspond to alternative transcript isoforms derived from the same genomic locus rather than to distinct gene copies, reflecting alternative splicing events and redundancy in the current transcript model annotation. However, for two candidates, *ACT* and *βTUB*, the BLAST-based transcript assignment suggested that mapped transcripts may originate from more than one genomic locus.

In general, genes represented by multiple gene copies are not ideal reference genes for qRT-PCR, as individual gene copies might by differentially regulated resulting in ambiguous transcript quantification. This might correlate with the fact that both *ACT* and *βTUB* were often found as moderately or least stable under the conditions of our experiment (Table 2). If this was the case, it constitutes a further rationale for excluding *ACT* and *βTUB* from use as reference genes for qRT-PCR analyses.

On the other hand, in plants, many genes with fundamental cellular functions or broad expression patterns are organized into multigene families, and disabled copies of these genes are often preserved as pseudogenes originating from duplicated paralogs [54,55,56,57]. This includes also the nuclear 35S/45S rDNA (encoding 18S-5.8S-26S rRNAs) which occurs as tandemly repeated arrays with hundreds to thousands of copies in plant genomes [58]. Despite that, 18S rRNA sequences are still being proposed as internal controls for molecular analyses in various plants, including species of medicinal importance [59,60,61,62].

In summary, for qRT-PCR normalization, it is generally recommended to prioritize single-copy genes with well-defined gene models and to empirically validate their stability under the specific experimental conditions.

The integrated RefFinder analysis provides practical, context-specific recommendations for reference gene selection. Its user-friendly interface and incorporation of multiple algorithms have contributed to its broad adoption. A key strength of RefFinder is its ability to generate a consensus ranking through a weighted geometric mean, thereby increasing confidence in identifying stable reference genes. However, as with other closed-source algorithms, RefFinder has several notable limitations, which we have described in detail in our previous publication on Reynoutria, the genus belonging to the Caryophyllales order and Polygonaceae family, therefore more distantly related than Caryophyllaceae family species [19]. Its opacity limits user customization, complicates troubleshooting, and may reduce suitability for certain datasets. Despite these caveats, RefFinder remains a valuable and widely used tool for strengthening qPCR normalization through multifactorial evaluation of reference gene stability [17,63,64,65,66,67].

One of the rarely studied factors that might also influence the baseline gene expression are diurnal or circadian fluctuation. In quinoa, a distant relative from the Amaranthaceae, two other genes—*Polypyrimidine tract-binding protein* (*PTB*) and *Isocitrate dehydrogenase* (*IDH-A*) were designated suitable as reference for diurnally regulated genes during a circadian cycle to avoid misinterpretation [68]. In our study, this issue has not been considered since the targeted genes were related to terpenoid pathway, while other potential targets represent phenylpropanoid pathway and RIP expression all of which that are rather stress and development associated than typical diurnally regulated genes. Nonetheless, to avoid additional variation, the plant material was collected during the morning hours.

In relation to the few previous studies on the other Caryophyllaceae, several genes can be suggested as preferred reference candidates, but the stability was not consistent under different experimental conditions and between the different tissues or genotypes. In *Silene vulgaris* [52] *SvACT* (*actin*) and *SvGAPDH* (*glyceraldehyde-3-phosphate dehydrogenase*) exhibited the lowest expression variability of the twenty genes across all tested tissues and organs, including pollen as well as between female and hermaphroditic plants. However, no varied conditions nor treatments were applied in the above study. Similarly, Rodriguez-Parra et al. [51] tested thirteen genes in *Dianthus broteri* organs and four ploidy levels and suggest that *TIP41* and *TIF5A* should be used when both petal and leaf tissues and various ploidy levels are to be compared. Unlike in *S. vulgaris*, *GAPDH* was unsuitable showing differential expression among cytotypes. In *Dianthus caryophyllus*, twelve candidate genes were tested by Yu et al. [50] in vegetative and generative organs as well as in leaves and roots under a set of stress treatments, such as: osmotic, salt, temperature, Cu^2+^ and Cr^3+^ ions, and exogenous plant growth regulators (auxin, cytokinin, ABA and gibberellin). The stability of candidate genes varied across tested tissues and treatments. As a result, several pairs of reference genes were recommended, depending on experimental conditions and organs, including *EF1α*, *UBQ1*, *CYP*, *TUB*, *TIP41*, *TIF5A*, *PP2A*. Hence, in carnations, each experimental set-up requires careful selection of the most suitable reference genes.

Much more studies have been published on more distant relatives, representing various families of the Caryophyllales order, such as Amaranthaceae, e.g., pseudocereals quinoa [13], amaranth [69] and several halophytes—*Halostachys*, *Salicornia*, *Salsola*, *Suaeda* [70,71,72,73] with the main aim of validating salt and drought stress response and developmental variation research. Again, these studies align with the main conclusion that there are no universal reference genes that would fit most needs in various experimental designs and different models.

Thus, even the closely related species can vary significantly in stability of candidate reference genes. This underscores the imperative for rigorous validation of candidate reference genes for each unique experimental setup and organism.

In conclusion, our study offers a thorough assessment of candidate reference genes for accurate qPCR normalization in *A. githago*. Reference gene stability varied markedly across plant organs and cultivation conditions, emphasizing the need for experiment-tailored selection of gene expression normalizers. By integrating multiple analytical approaches, we identified reference genes suitable for broad application as well as for conditions- and organ-specific normalization. These results will provide a basis for reliable gene expression studies in *A. githago* and support future research on the molecular regulation of secondary metabolism and stress responses in this medicinally important plant species.

## 4. Materials and Methods

### 4.1. Plant Material

Seeds of wild-type, open-pollinated *A. githago* were obtained from the certified collection of the Botanical Garden of Medicinal Plants at the Wroclaw Medical University, Poland (51.11736° N, 17.07486° E) operating under the Ministry of Environment accreditation No. DOPogiz-4210-26-6024-/05/kL).

The plants were grown in 15 diameter pots with soil, in glasshouse conditions. A voucher specimen with assigned number P-146 was deposited in the Botanical Garden herbarium.

In vitro cultures were initiated from surface-sterilized seeds treated with 5% sodium hypochlorite, rinsed three times with distilled water. Seeds were sown on basal Murashige and Skoog (MS) medium [74] supplemented with 3% (*w*/*v*) sucrose and solidified with 6 g/L agar (Biocorp, Warsaw, Poland).

Shoot tips of the three-weeks-old aseptic seedlings containing two nodes were excised and transferred to proliferation media supplemented with phytohormones or elicitors of biotic or abiotic stress at varying concentrations. Salicylic acid (SA) or methyl jasmonate (MeJa) were applied at 100 μM and 250 μM; yeast extract (YE) at 250 mg/L and 1000 mg/L; and mannitol at 100 μM and 800 μM. Shoots cultured on basal MS medium without supplementation served as controls.

Cultures were maintained in a growth chamber (POL-EKO, Wodzisław Śląski, Poland) at 25 ± 2 °C under a 16/8 h light/dark photoperiod using white LED lamps (8000–10,000 K) at a photon flux density of 55 μmol/m^2^s, with 40% relative humidity.

Aerial parts of the plants were harvested after 7, 14, and 28 days of stress treatment, except for MeJa-treated shoots, which did not survive until the final sampling. In total, 25 distinct *A. githago* samples were collected, representing four stress types, two concentrations each, and three time points, including controls. Each sample comprised six biological replicates. All samples were immediately frozen in liquid nitrogen, homogenized, and subsequently stored at −80 °C for future experiments.

Glasshouse experiment was carried out in Botanical Garden of Medicinal Plants Wroclaw Medical University. Plants were cultivated in pots (15 × 15 cm; 11 L) filled with commercially available soil composed of 85% of organic matter, 2.5 kg/m^3^ organic fertilizers and 1.25 kg/m^3^ mineral fertilizers (Canna Terra Professional containing NPK:12/14/24, pH 6.0, EC: 1.1–1.3 mS/cm, product code: 37E5-305D1, CANNA Nederland BV, Breda, The Netherlands) and watered daily. Growing parameters including air and soil temperature, soil pH and air humidity were monitored and recorded daily and are provided in the Supplementary Table S5.

*A. githago* plants were harvested based on organ type: roots, leaves, flowers and fruits at seven time-points covering the developmental stages throughout the vegetative season. Samples from six individualy grown plants were collected for a study, at each time-point. In total, 15 distinct soil-grown *A. githago* samples representing different organs and developmental stages were collected. The plant samples were collected during morning hours (between 9 and 11 a.m.). Samples were immediately frozen in liquid nitrogen, homogenized, and subsequently stored at −80 °C for future experiments.

The sample list with detailed description of cultivation type, plant organ and stress conditions as well as a harvest time-point is provided in the Supplementary Table S6.

### 4.2. RNA Extraction and cDNA Synthesis

Total RNA was extracted using the Plant/Fungi Total RNA Purification Kit (Norgen Biotek Corp., Thorold, ON, Canada) from 50 mg of each sample. RNA purification with DNase treatment (RNase-Free DNase I, Norgen Biotek Corp., Thorold, ON, Canada) was performed using an on-column workflow according to the manufacturer’s protocol. The extracted RNA was assessed for quality and purity by the NanoDrop 2000 spectrophotometer (Thermo Fisher Scientific^TM^, Waltham, MA, USA). RNA samples with an A260/A280 ratio of 1.9–2.1 and an A260/A230 ratio > 2.0 were used for subsequent cDNA synthesis. RNA was stored at –80 °C to maintain its quality and integrity.

Each cDNA was synthesized from 5 µg of total RNA in a total volume of 40 µL using the iScript^TM^ Advanced cDNA Synthesis Kit for qRT-PCR (Bio-Rad, Hercules, CA, USA). The initial reaction comprised: 5 µg of total RNA, 8 µL of 5× iScript^TM^ Advanced Reaction Mix (Bio-Rad, Hercules, CA, USA) (which includes dNTPs, oligo(dT), and random primers), 2 µL of iScript^TM^ Advanced Reverse Transcriptase (Bio-Rad, Hercules, CA, USA), and nuclease-free water. This mixture was incubated at 46 °C for 20 min, followed by a heating step at 95 °C for 1 min. Subsequently, each cDNA was diluted five times with nuclease-free water to maintain the same range of template concentrations across all samples.

### 4.3. Selection of Candidate Reference Genes, Genes Related to Saponin Biosynthesis and Primer Design

Seven candidate reference genes—*actin*, *β-tubulin*, *elongation factor 1α*, *glyceraldehyde-3-phosphate dehydrogenase*, *histone H3*, *translation elongation factor 1*, and *eukaryotic translation initiation factor 5A1,* were selected based on previous studies in carnation species: *Dianthus caryophyllus* [50] and *Dianthus broteri* [51].

Nucleotide sequences of these genes were retrieved from the transcriptomic data of *A. githago* [32]. To confirm gene identity and link transcriptome-derived contigs to the reference genome, we performed BLASTN searches against a custom cDNA database derived from the annotated *A. githago* reference genome (assembly dcAgrGith1.1) generated within the Darwin Tree of Life project (Ensembl Darwin Tree of Life portal; https://projects.ensembl.org/darwin-tree-of-life/, accessed on 20 December 2025) [36].

All *A. githago* RNA sequences used in this study were queried with BLASTN (BLAST+ v2.15.0) against the cDNA database, allowing up to 50 target sequences per query (max_target_seqs = 50). For each query, tabular BLAST outputs (outfmt 6: qseqid, sseqid, percentage identity, alignment length, number of mismatches and gaps, query and subject start/end positions, E-value, bitscore and subject annotation) were generated and sorted to select either the single best hit or up to the five highest-scoring hits per sequence. Ranking was based primarily on percentage identity, with bitscore and E-value. In parallel, full BLAST reports (outfmt 0) were produced for the same hit sets to allow inspection of complete pairwise alignments and coverage. BLAST annotation was performed against public NCBI databases without taxonomy restriction (i.e., across all organisms).

The resulting contigs with their BLAST score parameters and nucleotide sequences are provided in the Supplementary Table S7.

Primer pairs were designed using Primer3Plus (https://www.primer3plus.com/, accessed on 21 April 2024) [75]. Primer design parameters were set to a melting temperature of 60 °C, length of 20–22 bp, and ~50% GC content (Table 1). Prior to qRT-PCR assays, each primer pair’s amplification efficiency (E) and specificity were assessed using a ten-fold cDNA dilution series to generate standard curves. Efficiency was calculated as E [%] = (10^−1/slope^ − 1) × 100% [76].

### 4.4. Quantitative Real-Time PCR

Gene-specific primers (Table 1) were employed to quantify transcript levels from reverse-transcribed cDNA templates. Reactions were carried out in triplicate with a CFX96 Touch Real-Time PCR Detection System (Bio-Rad) using SsoAdvanced^TM^ Universal SYBR Green Supermix^®^ (Bio-Rad, Hercules, CA, USA). The reaction mixture consisted of 5 μL of SsoAdvanced Universal SYBR^®^ Green Supermix (2×), 2 μL of nuclease-free H_2_O, 1 μL of previously diluted cDNA, and 2 μL of a mix of forward and reverse amplification primers (250 nmol/μL each), resulting in a final volume of 10 μL.

Thermal cycling comprised an initial denaturation at 95 °C for 30 s, followed by 40 cycles of 95 °C for 15 s and 60 °C for 30 s. A subsequent melt-curve analysis was performed from 65 °C to 95 °C at a rate of 0.5 °C per 5 s to verify amplicon specificity.

Primer efficiencies were determined experimentally from a ten-fold cDNA dilution series and incorporated into relative quantification calculations via the Pfaffl method [77]. All stages, from experimental design and RNA isolation through data analysis, complied with the MIQE guidelines for qRT-PCR [5]. Relative expression values are provided in Supplementary Table S1.

### 4.5. Statistical Data Analysis

Several commonly used algorithms, including ΔCt, BestKeeper, GeNorm, NormFinder, and a web-based tool RefFinder (https://www.ciidirsinaloa.com.mx/RefFinder-master/, 20 October 2025), were applied to assess the stability of candidate reference genes [17,66,78] in various experimental conditions and organs of *A. githago*. GeNorm and NormFinder use the 2^−ΔCT^ method for calculations, while BestKeeper analyzes the Ct values. GeNorm calculates the stability of a reference gene (M) based on the average pairwise variations (V) among all other reference genes. It also determines the optimal number of reference genes needed by computing the Vn/Vn+1 pairwise variation. The NormFinder algorithm evaluates both intra- and intergroup expression variations, assigning the highest stability to genes with the lowest stability values (SV). The BestKeeper program evaluates the expression stability of genes by assessing coefficients of variation (CV) and standard deviations (SD), with the lowest CV and SD used as criteria for identifying the most stable reference genes. Lastly, the results from the above algorithms are combined by the web-based analysis tool RefFinder. RefFinder is an online tool that brings together the results of major computational programs, including GeNorm (M values), NormFinder (stability values), BestKeeper (CV and SD), and ΔCt values. It calculates the overall expression stability ranking by taking the geometric mean of all rankings and generates a comprehensive reference gene stability ranking, which helps choose the most suitable reference genes. Using a weighted geometric mean–based integration of rankings from ΔCt, geNorm, NormFinder, and BestKeeper reduces bias associated with any single algorithm, provides a consensus ranking that reflects multiple statistical perspectives and improves confidence in selecting the most stable reference genes.

### 4.6. Validation of Selected Reference Genes

Using the selected reference genes, we further analyzed the expression patterns of *squalene synthase* (*SQS*) and *β-amyrin synthase* (*BAS*) across selected experimental stress conditions and various organs of soil-grown *A. githago* to validate the stability of these reference genes. The samples and procedures remained the same as those used in the qPCR analysis of the reference genes. We assessed the relative expression level using the calculated primer efficiency [77].

## 5. Conclusions

To enable robust analyses of genes involved in natural product biosynthesis and specialized metabolic regulation in *A. githago*, we determined the most stably expressed reference genes across multiple plant organs and cultivation conditions in this species.

Based on the overall ranking, we recommend *TIF5A1-2* and *GAPDH* as suitable reference genes for in vitro experiments, and *EF1α* together with *H3* for studies involving different organs and developmental stages of soil-grown *A. githago*. For universal normalization across all tested organs, developmental stages, and cultivation conditions, the combination of *H3* and *TIF5A1-2* is proposed, as these genes showed the highest expression stability.

## Figures and Tables

**Table 1:** qPCR primers used in this study.

Primer Name	Left Primer Sequence 5′ → 3′	Right Primer Sequence 5′ → 3′	Product Length [bp]	Efficiency [%]	R^2^
*Actin (ACT)*	CTGTGTGGCTCACACCATCT	ACTTTCAATGCGCCTGCTAT	112	108	0.998
*β-Tubulin (βTUB)*	AGAAGTGAAGTCGGGGGAAT	AACCACTTGATCTCGGCAAC	121	105	0.999
*Elongation factor 1α (EF1α)*	CCACAACCACTGGTCACTTG	GTTCGGCCTTAAGCTTGTCA	138	99	0.996
*Glyceraldehyde-3-phosphate dehydrogenase (GAPDH)*	AAGGTCTTGCCAGCTTTGAA	TAGGTTGCAGCCTTCTCGAT	110	110	0.993
*Histone H3 (H3)*	GTGAAGAAGCCCCACAGGTA	CTGGAATGGGAGTTTCCTGA	101	96	0.999
*Translation elongation factor 1 (TEF1)*	GTTTTTGTGATGCGTGATGC	GAAGGCTTCAGACCAAGACG	149	101	0.995
*Eukaryotic translation initiation factor 5A1 (TIF5A1-1)*	GTCGGACGAAGAACACCAAT	GCGATTTTTGATGACGAGGT	112	117	0.992
*Eukaryotic translation initiation factor 5A1 (TIF5A1-2)*	AATGGCAAGAAGCTTGAGGA	GCAGACTAACGAAGCCATCC	115	113	0.991
*Squalene synthase (SQS)*	TGGCACTGAACTTCGCAATG	AACATCCGTGGCTATGCTTG	94	114	0.997
*β-amyrin synthase (BAS)*	ATCGCCGAGGATGGACTTAAG	TTCGAGGCAATCAACGCTTG	86	112	0.997

**Table 2:** Comprehensive stability rankings of candidate reference genes as determined by RefFinder across individual treatments and growth conditions.

		Ranking Order (from the Most Stable to the Least Stable Gene)							
Treatment and Growth Conditions	Method	1	2	3	4	5	6	7	8
Salicylic acid									
	ΔCT	*TIF5A1-1*	*GAPDH*	*H3*	*TIF5A1-2*	*EF1α*	*ACT*	*TEF1*	*βTUB*
	BestKeeper	*TIF5A1-2*	*H3*	*GAPDH*	*TIF5A1-1*	*EF1α*	*TEF1*	*ACT*	*βTUB*
	NormFinder	*GAPDH*	*TIF5A1-1*	*EF1α*	*ACT*	*H3*	*TIF5A1-2*	*TEF1*	*βTUB*
	GeNorm	*H3|TIF5A1-2*		*TIF5A1-1*	*GAPDH*	*EF1α*	*ACT*	*TEF1*	*βTUB*
	Comprehensive ranking	*TIF5A1-1*	*GAPDH*	*TIF5A1-2*	*H3*	*EF1α*	*ACT*	*TEF1*	*βTUB*
MeJA									
	ΔCT	*H3*	*TIF5A1-2*	*ACT*	*GAPDH*	*TIF5A1-1*	*EF1α*	*TEF1*	*βTUB*
	BestKeeper	*H3*	*TIF5A1-2*	*EF1α*	*GAPDH*	*TEF1*	*ACT*	*TIF5A1-1*	*βTUB*
	NormFinder	*TIF5A1-1*	*ACT*	*H3*	*GAPDH*	*TIF5A1-2*	*EF1α*	*TEF1*	*βTUB*
	GeNorm	*H3 | TIF5A1-2*		*GAPDH*	*EF1α*	*ACT*	*TIF5A1-1*	*TEF1*	*βTUB*
	Comprehensive ranking	*H3*	*TIF5A1-2*	*ACT*	*GAPDH*	*TIF5A1-1*	*EF1α*	*TEF1*	*βTUB*
Yeast extract									
	ΔCT	*GAPDH*	*TIF5A1-2*	*ACT*	*H3*	*TIF5A1-1*	*EF1α*	*TEF1*	*βTUB*
	BestKeeper	*EF1α*	*GAPDH*	*ACT*	*βTUB*	*TEF1*	*TIF5A1-2*	*H3*	*TIF5A1-1*
	NormFinder	*GAPDH*	*TIF5A1-2*	*ACT*	*EF1α*	*H3*	*TIF5A1-1*	*TEF1*	*βTUB*
	GeNorm	*H3 | TIF5A1-1*		*TIF5A1-2*	*GAPDH*	*ACT*	*EF1α*	*TEF1*	*βTUB*
	Comprehensive ranking	*GAPDH*	*TIF5A1-2*	*ACT*	*H3*	*EF1α*	*TIF5A1-1*	*TEF1*	*βTUB*
Mannitol									
	ΔCT	*TIF5A1-2*	*GAPDH*	*TIF5A1-1*	*ACT*	*H3*	*EF1α*	*TEF1*	*βTUB*
	BestKeeper	*H3*	*TIF5A1-2*	*EF1α*	*GAPDH*	*TEF1*	*TIF5A1-1*	*ACT*	*βTUB*
	NormFinder	*ACT*	*GAPDH*	*TIF5A1-1*	*TIF5A1-2*	*EF1α*	*H3*	*TEF1*	*βTUB*
	GeNorm	*GAPDH | TIF5A1-2*		*H3*	*TIF5A1-1*	*EF1α*	*ACT*	*TEF1*	*βTUB*
	Comprehensive ranking	*TIF5A1-2*	*GAPDH*	*H3*	*ACT*	*TIF5A1-1*	*EF1α*	*TEF1*	*βTUB*
Leaves									
	ΔCT	*EF1α*	*βTUB*	*ACT*	*GAPDH*	*H3*	*TIF5A1-2*	*TEF1*	*TIF5A1-1*
	BestKeeper	*H3*	*TIF5A1-2*	*TIF5A1-1*	*βTUB*	*EF1α*	*GAPDH*	*ACT*	*TEF1*
	NormFinder	*βTUB*	*EF1α*	*ACT*	*H3*	*GAPDH*	*TIF5A1-2*	*TEF1*	*TIF5A1-1*
	GeNorm	*EF1α | GAPDH*		*ACT*	*βTUB*	*TEF1*	*H3*	*TIF5A1-2*	*TIF5A1-1*
	Comprehensive ranking	*EF1α*	*βTUB*	*GAPDH*	*H3*	*ACT*	*TIF5A1-2*	*TIF5A1-1*	*TEF1*
Roots									
	ΔCT	*TIF5A1-2*	*TIF5A1-1*	*H3*	*GAPDH*	*βTUB*	*ACT*	*EF1α*	*TEF1*
	BestKeeper	*EF1α*	*H3*	*GAPDH*	*TIF5A1-2*	*TIF5A1-1*	*TEF1*	*βTUB*	*ACT*
	NormFinder	*TIF5A1-2*	*TIF5A1-1*	*H3*	*GAPDH*	*βTUB*	*EF1α*	*ACT*	*TEF1*
	GeNorm	*ACT | βTUB*		*TIF5A1-1*	*TIF5A1-2*	*GAPDH*	*H3*	*EF1α*	*TEF1*
	Comprehensive ranking	*TIF5A1-2*	*TIF5A1-1*	*H3*	*βTUB*	*GAPDH*	*EF1α*	*ACT*	*TEF1*
Reproductive organs									
	ΔCT	*TIF5A1-1*	*TIF5A1-2*	*EF1α*	*H3*	*GAPDH*	*βTUB*	*ACT*	*TEF1*
	BestKeeper	*TIF5A1-1*	*TIF5A1-2*	*βTUB*	*GAPDH*	*ACT*	*EF1α*	*H3*	*TEF1*
	NormFinder	*TIF5A1-1*	*TIF5A1-2*	*H3*	*EF1α*	*GAPDH*	*βTUB*	*ACT*	*TEF1*
	GeNorm	*TIF5A1-1 | TIF5A1-2*		*H3*	*EF1α*	*GAPDH*	*βTUB*	*ACT*	*TEF1*
	Comprehensive ranking	*TIF5A1-1*	*TIF5A1-2*	*H3*	*EF1α*	*GAPDH*	*βTUB*	*ACT*	*TEF1*
In vitro cultures									
	ΔCT	*GAPDH*	*TIF5A1-2*	*TIF5A1-1*	*H3*	*ACT*	*EF1α*	*TEF1*	*βTUB*
	BestKeeper	*H3*	*TIF5A1-2*	*GAPDH*	*TEF1*	*TIF5A1-1*	*EF1α*	*ACT*	*βTUB*
	NormFinder	*GAPDH*	*TIF5A1-2*	*TIF5A1-1*	*ACT*	*EF1α*	*H3*	*TEF1*	*βTUB*
	GeNorm	*H3 | TIF5A1-2*		*GAPDH*	*TIF5A1-1*	*EF1α*	*ACT*	*TEF1*	*βTUB*
	Comprehensive ranking	*TIF5A1-2*	*GAPDH*	*H3*	*TIF5A1-1*	*ACT*	*EF1α*	*TEF1*	*βTUB*
Soil cultures									
	ΔCT	*EF1α*	*H3*	*βTUB*	*TIF5A1-2*	*TIF5A1-1*	*TEF1*	*ACT*	*GAPDH*
	BestKeeper	*H3*	*EF1α*	*TIF5A1-2*	*TIF5A1-1*	*βTUB*	*GAPDH*	*ACT*	*TEF1*
	NormFinder	*EF1α*	*H3*	*βTUB*	*TIF5A1-2*	*TIF5A1-1*	*TEF1*	*ACT*	*GAPDH*
	GeNorm	*TIF5A1-1 | TIF5A1-2*		*H3*	*EF1α*	*βTUB*	*TEF1*	*ACT*	*GAPDH*
	Comprehensive ranking	*EF1α*	*H3*	*TIF5A1-2*	*TIF5A1-1*	*βTUB*	*TEF1*	*ACT*	*GAPDH*
All samples									
	ΔCT	*H3*	*TIF5A1-2*	*EF1α*	*TIF5A1-1*	*ACT*	*GAPDH*	*βTUB*	*TEF1*
	BestKeeper	*H3*	*TIF5A1-2*	*EF1α*	*GAPDH*	*TIF5A1-1*	*TEF1*	*ACT*	*βTUB*
	NormFinder	*EF1α*	*H3*	*TIF5A1-2*	*TIF5A1-1*	*GAPDH*	*ACT*	*βTUB*	*TEF1*
	GeNorm	*TIF5A1-1 | TIF5A1-2*		*H3*	*EF1α*	*GAPDH*	*ACT*	*βTUB*	*TEF1*
	Comprehensive ranking	*H3*	*TIF5A1-2*	*EF1α*	*TIF5A1-1*	*GAPDH*	*ACT*	*βTUB*	*TEF1*

## Data Availability

Data generated and analyzed during this study are included in this published article and its Supplementary Information Files.

## Supplementary Materials

The following supporting information can be downloaded at https://www.mdpi.com/article/10.3390/ijms27020889/s1.

## Abbreviations

The following abbreviations are used in this manuscript:

*ACT*	*actin*
ANOVA	analysis of variance
BAS	*β-amyrin synthase*
Ct	cycle threshold
CV	coefficient of variation
D	day of treatment
E	amplification efficiency
*EF1α*	*elongation factor 1α*
*GAPDH*	*glyceraldehyde-3-phosphate dehydrogenase*
H	harvest
*H3*	*histone H3*
MeJA	methyl jasmonate
MN	mannitol
qRT-PCR	quantitative real-time polymerase chain reaction
R^2^	correlation coefficient
RIP	ribosome-inactivating proteins
SA	salicylic acid
SD	standard deviation
SQS	*squalene synthase*
*TEF1*	*translation elongation factor 1*
*TIF5A1*	*eukaryotic translation initiation factor 5A1*
YE	yeast extract
*β-TUB*	*β-tubulin*
